# Effectiveness and Safety of Dose-Specific DOACs in Patients With Atrial Fibrillation: A Systematic Review and Network Meta-Analysis

**DOI:** 10.1155/cdr/9923772

**Published:** 2025-01-06

**Authors:** Sang-Hyeon Oh, Seunghyun Cheon, Seo-Yong Choi, Young Seo Kim, Han-Gon Choi, Jee-Eun Chung

**Affiliations:** ^1^College of Pharmacy and Institute of Pharmaceutical Science and Technology, Hanyang University, Ansan-si, Gyeonggi-do, Republic of Korea; ^2^Department of Neurology, College of Medicine, Hanyang University, Seoul, Republic of Korea

**Keywords:** atrial fibrillation, DOAC, low dose, major bleeding, meta-analysis, stroke

## Abstract

**Background:** Dose adjustments of direct-acting oral anticoagulants (DOACs) for atrial fibrillation are based on pivotal clinical trials assessing their effectiveness and safety in controlled settings. However, the appropriateness of these dosing strategies in real-world practice is uncertain. The purpose of this study is to compare the effectiveness and safety of dose-specific DOACs with those of warfarin.

**Methods:** This study retrieved articles from MEDLINE, Embase, and CENTRAL until March 5, 2024. Primary outcomes were the incidence of stroke/systemic embolisms (S/SEs) and major bleeding (MB). Direct pairwise meta-analyses compared each dose-specific DOAC with warfarin. Heterogeneity was assessed using Higgin's *I*^2^ and *Q* statistics, while publication bias was evaluated through funnel plots and Begg's and Egger's tests, with adjusted pooled estimates calculated via trim-and-fill and precision-effect estimate with standard error (PET-PEESE) methods. A network analysis was conducted, with additional comparisons made using a Bayesian random-effects model for indirect evidence.

**Results:** A total of 32 studies with 2,332,770 patients were included. Both standard-dose (SD) and low-dose (LD) DOACs significantly reduced S/SE, except for LD apixaban and LD edoxaban. Rivaroxaban did not show significant difference in MB compared to warfarin. In East Asian patients, all doses of DOACs exhibited lower hazard ratios (HRs) for S/SE and MB than those observed in the primary analysis, with LD rivaroxaban significantly reducing MB, a finding not observed in the primary analysis. Rank probability analysis indicated that the dose-specific DOACs had different safety profiles and small but meaningful differences in effectiveness. SD apixaban (S/SE: second, MB: second) and edoxaban (S/SE: first, MB: fourth) and LD edoxaban (S/SE: fourth, MB: first) had high ranks. LD apixaban had the most significant difference in rank for S/SE from SD apixaban, ranking eighth compared to second.

**Conclusions:** This study found that all DOACs provided comparable or superior effectiveness and safety to warfarin. SD apixaban, SD edoxaban, and LD edoxaban achieved a favorable balance between preventing S/SE and MB risk.

## 1. Introduction

Atrial fibrillation (AF), a prevalent cardiac arrhythmia, significantly elevates the risk of ischemic stroke and thromboembolism. To prevent these life-threatening events, direct-acting oral anticoagulants (DOACs) have emerged as the preferred therapeutic option for AF patients due to their noninferior effectiveness, superior safety profile, and enhanced convenience compared to warfarin [1, 2].

While DOACs offer simplified dosing regimens, personalized dose adjustments may be necessary based on specific clinical factors. Patients with reduced kidney function often require lower dosages for certain DOACs, such as dabigatran and rivaroxaban. Regarding body weight, lower body weight may necessitate adjusted dosages, particularly with apixaban and edoxaban. Patients with advanced age (> 80 years) require dose reductions across various DOACs. Regarding concomitant medications, interactions with certain drugs may necessitate DOAC dose adjustments [3–7].

Current prescribing guidelines specify dose adjustments for these factors. The doses of apixaban and edoxaban are recommended to be reduced by 50%, while the doses of dabigatran and rivaroxaban are recommended to be reduced by approximately 25% [3]. However, a concerning practice gap exists, particularly in East Asia, where physicians often favor prescribing lower-than-recommended DOAC doses regardless of individual patient characteristics [8]. Compared to non-Asian patients, Asian patients with AF frequently present with advanced age, medical fragility, and lower body mass, which may increase their susceptibility to adverse drug effects at the same dose [9]. Although concerns regarding the risk of bleeding in East Asian patients may contribute to this preference for low dose, undertreating with reduced DOAC doses could compromise their effectiveness in preventing stroke/systemic embolisms (S/SEs) [10, 11]. It is clinically significant to achieve an appropriate balance between the bleeding risk and the potential for inadequate S/SE prevention. The US Food and Drug Administration has emphasized the potential harms of inadequate anticoagulation for stroke prevention [12].

Given the conflicting evidence and concerns surrounding DOAC dosing practices, this study is aimed at comparing the risk of S/SE and major bleeding (MB) for each dosing regimen in AF patients taking DOAC through a systematic review and network meta-analysis.

## 2. Methods

### 2.1. Search Strategy

The search included the following keywords: atrial fibrillation, AF, apixaban, dabigatran, rivaroxaban, edoxaban, direct oral anticoagulants, DOACs, novel oral anticoagulants, NOACs, warfarin, vitamin K antagonist, VKA, stroke, cerebral infarction, TIA, ischemic heart disease, myocardial infarction, acute coronary syndrome, thromboembolic event, MB, and their synonyms and related keywords. The primary literature search was conducted with literature published up to August 15, 2022, after which a secondary literature search was conducted to include very recent articles. Therefore, articles retrieved from MEDLINE, Embase, and CENTRAL up to March 5, 2024, were included in this study. After removing duplicates, two researchers, Sang-Hyeon Oh and Seunghyun Cheon, independently screened the titles and abstracts of all records to identify potentially eligible studies. A full-text review was subsequently conducted to determine final inclusion according to the eligibility criteria. In instances of disagreement, a consensus was reached through discussion, with the involvement of a third expert, Jee-Eun Chung, to facilitate the review process. The implementation and reporting of systematic literature reviews followed the Preferred Reporting Items for Systematic Reviews and Meta-Analyses (PRISMA) statement (Table S1) [13]. This study protocol was registered with the International Platform of Registered Systematic Review and Meta-Analysis Protocols (Registration Number INPLASY202280073).

The following inclusion criteria were used: (1) provided dosing regimen for DOACs; (2) provided sufficient information to use a hazard ratio (HR) and 95% confidence interval (CI). Exclusion criteria were (1) reviews, comments, letters, news, case reports, case series, surveys, guidelines, and editorials; (2) studies without appropriate outcomes, including those with no dose-specific result, no data required to calculate HR, or unclear definitions of S/SE and MB events; (3) studies that did not compare DOAC with warfarin; and (4) repeated study populations.

### 2.2. Data Extraction

The following information was extracted from each study: name of the first author, publication year, country of study, study design, number of patients per treatment arm, patient age and gender, follow-up period, drug regimen, and HR with a 95% CI for outcomes. Additionally, the CHA_2_DS_2_-VASc and HAS-BLED scores were extracted, as they encompass essential information on comorbid cardiovascular conditions, such as congestive heart failure, hypertension, and diabetes mellitus, as well as the concurrent use of antiplatelet agents [14]. These scores serve as potential confounders, offering a comprehensive interpretation of differences in outcomes across studies by accounting for variations in patient characteristics. Each DOAC was stratified by dose (standard dose (SD) and low dose (LD)), and interventions of this study were divided into eight groups: four for SD users (apixaban 5 mg bid, dabigatran 150 mg bid, edoxaban 60 mg qd, rivaroxaban 20 mg qd) and four for LD users (apixaban 2.5 mg bid, dabigatran 110 mg bid, edoxaban 30/15 mg qd, rivaroxaban 15 mg qd). The primary outcomes of S/SE and MB were extracted for each group. All-cause mortality that occurred during the follow-up period of each study was evaluated as the secondary outcome.

### 2.3. Quality Assessment

To evaluate the methodological quality of each study, we used the tools recommended by the Cochrane Association for bias assessments. Risk of bias by nonrandomized studies (RoBANS) for cohort studies and Cochrane's assessment of the risk of bias (RoB) for randomized controlled trials (RCTs) were used (Figure S1) [15]. Publication bias was assessed by funnel plots, Begg's test, and Egger's test. We also calculated adjusted pooled estimates to reflect the impact of missing studies estimated by the trim-and-fill and precision-effect estimate with standard error (PET-PEESE) methods. After that, the impact of publication bias on the degree of change in original pooled estimates was examined. Assessment of publication bias was performed using the metafor package (3.0-2 version) of R software (4.1.0 version).

### 2.4. Statistical Analysis

The primary outcome was comparing the incidence of S/SE or MB at specific dose of DOACs and that of warfarin. The secondary outcomes were all-cause mortality. The effect sizes were analyzed and expressed as HRs with 95% CIs for each study. The comparative study of specific-dose DOAC with warfarin was performed through a direct pairwise meta-analysis. The heterogeneity among the included studies was analyzed using Higgin's *I*^2^ statistics and *Q* statistics [16]. Additionally, further analysis was conducted excluding RCTs to investigate the results in real-world clinical practice. Sensitivity analysis was performed for studies with East Asian patients and with long-term (≥ 6 months) follow-up. The direct pairwise meta-analyses were performed in Review Manager 5.4. A Bayesian network model based on the Markov chain Monte Carlo (MCMC) operation was used to analyze the therapeutic effects of drugs in multiple groups [17]. To account for the correlation of effect estimates in multiarm trials, we assumed that the standard error was proportional to 1/√N  [18]. The random-effects model was used to calculate the effect size for the entire network meta-analysis. The MCMC simulation was built with four chains and set the burn-in period to 5000 iterations for each chain, followed by 20,000 iterations. The sample was obtained by extracting every 10th iteration. Convergence of the model was diagnosed through trace plot, density plot, and the potential scale reduction factor. The treatment ranking was expressed as a rank probability to evaluate the most effective treatment in the network meta-analysis. For interpretation, rank probability values range between 0 and 1, and values nearer to 1 are preferred. The network meta-analysis was performed using the GeMTC package (1.0-1 version) and Rjags package (4-10 version) of R software (4.1.0 version).

## 3. Results

### 3.1. Study Selection and Characteristics

**Figure 1:**
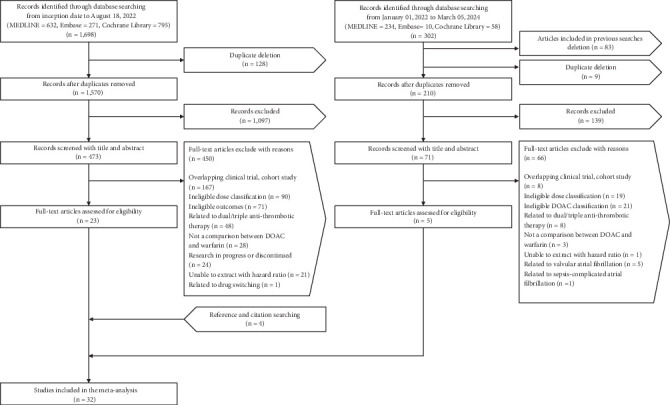
Overview of the study selection process.

The primary search identified a total of 1698 articles across three databases, from which 128 duplicates were removed. After eliminating 1097 articles during the title and abstract screening, we selected 473 studies for full-text review. Of these, 450 were excluded, and 23 studies that met the eligibility criteria were included. A second search identified an additional 302 articles. After removing articles included in previous searches, duplicates, and those filtered out during abstract screening, 65 full-text studies were reviewed. Five eligible articles were selected, and four additional studies were included based on references and citations. Ultimately, 32 studies involving 2,332,770 patients were included in the meta-analysis (Figure 1).  Table 1 describes the characteristics of the included studies. Of the 32 studies, six were RCTs, and 26 were cohort studies. Thirteen studies were conducted in East Asian populations, and 22 studies had long-term follow-up periods exceeding 6 months. Regarding DOACs, four studies focused exclusively on dabigatran [19, 25, 26, 29], six studies examined only rivaroxaban [21, 22, 24, 32, 36, 48], four studies investigated only apixaban [20, 42, 47, 50], and two studies analyzed only edoxaban [23, 33]. Of the 10 studies that included the three drugs—apixaban, dabigatran, and rivaroxaban—four were conducted in Europe [27, 30, 43, 44], four in North America [28, 31, 35, 45], and two in East Asia [34, 41]. One study conducted in Taiwan included three drugs: apixaban, dabigatran, and edoxaban [40]. Additionally, three studies incorporated all four DOACs and were conducted in East Asia [37–39], and the remaining two studies included two of the four DOACs [46, 49]. In all studies, the control drug was warfarin. The number of studies that included each DOAC was as follows: apixaban (*n* = 19), dabigatran (*n* = 19), edoxaban (*n* = 6), and rivaroxaban (*n* = 21). The number of patients in each study ranged from 313 to 501,990, with the mean or median age ranging from 67.6 to 85.6 years. The total number of studies and patients for a specific dose of DOACs is shown in Table S2.

### 3.2. Effectiveness and Safety

**Figure 2:**
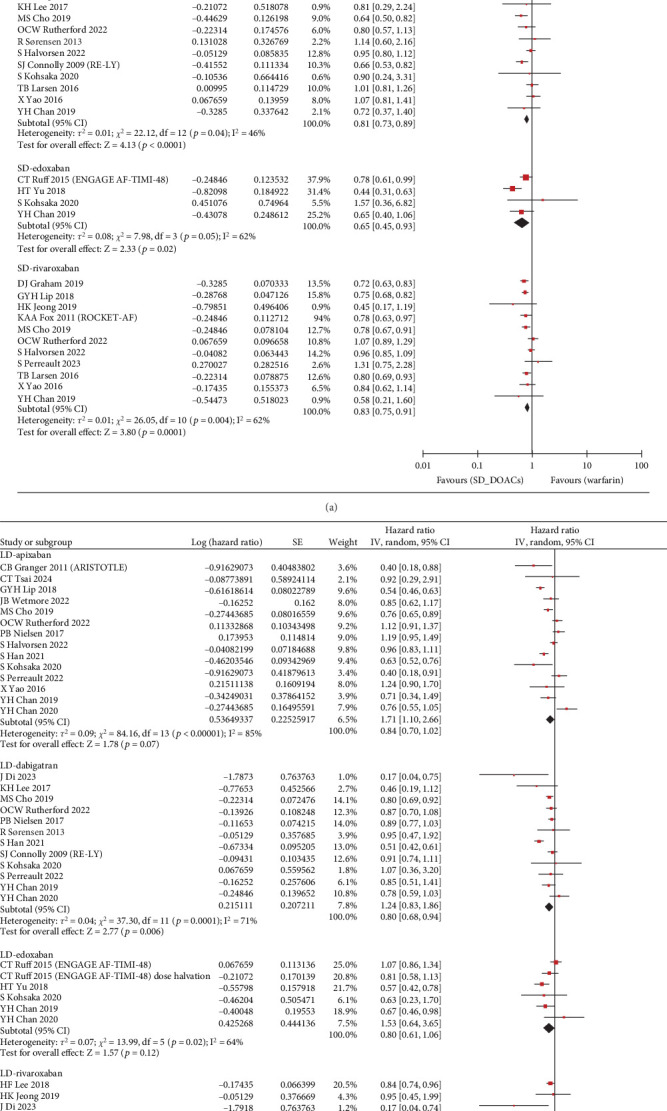
Forest plot of hazard ratio for stroke/systemic embolism: (a) standard dose; (b) low dose.

**Figure 3:**
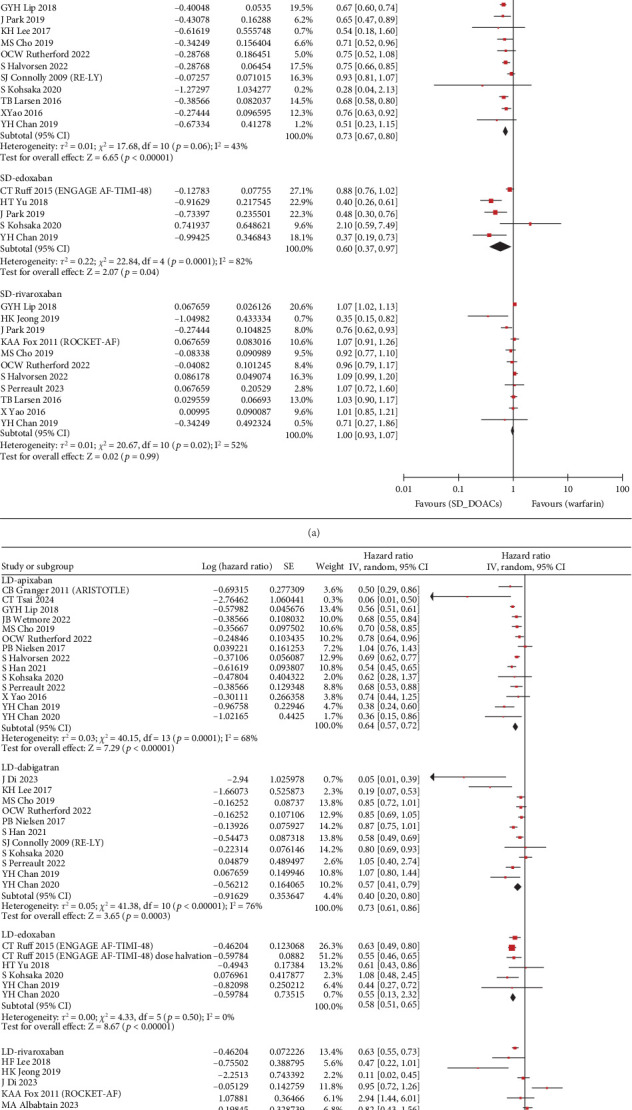
Forest plot of hazard ratio for major bleeding: (a) standard dose; (b) low dose.

The S/SE outcome was available in a total of 28 studies and a total of 1,789,446 patients. Compared with warfarin, the incidence of S/SE was significantly lower in patients taking SD apixaban (HR, 0.76; 95% CI, 0.67–0.86; *I*^2^, 73%), SD dabigatran (HR, 0.81; 95% CI, 0.73–0.89; *I*^2^, 46%), SD edoxaban (HR, 0.65; 95% CI, 0.45–0.93; *I*^2^, 62%), SD rivaroxaban (HR, 0.83; 95% CI, 0.75–0.91; *I*^2^, 62%), LD dabigatran (HR, 0.80; 95% CI, 0.68–0.94; *I*^2^, 71%), and LD rivaroxaban (HR, 0.82; 95% CI, 0.69–0.97; *I*^2^, 60%). However, the patients with taking LD apixaban or LD edoxaban had no statistically significant difference in S/SE incidence with warfarin users (Figure 2). Across 29 studies covering 1,742,404 patients, MB occurred more frequently in warfarin users than in patients taking SD apixaban (HR, 0.63; 95% CI, 0.58–0.69; *I*^2^, 74%), SD dabigatran (HR, 0.73; 95% CI, 0.67–0.80; *I*^2^, 43%), SD edoxaban (HR, 0.60; 95% CI, 0.37–0.97; *I*^2^, 82%), LD apixaban (HR, 0.64; 95% CI, 0.57–0.72; *I*^2^, 68%), LD dabigatran (HR, 0.73; 95% CI, 0.61–0.86; *I*^2^, 76%), and LD edoxaban (HR, 0.58; 95% CI, 0.51–0.65; *I*^2^, 0%). However, the patients taking rivaroxaban at any dose did not differ significantly in incidence of MB from warfarin users (Figure 3). In summary, the effectiveness and safety of SD apixaban, SD rivaroxaban, and both doses of dabigatran were superior to those of warfarin, making them preferable options for all patients; LD apixaban and LD edoxaban showed a lower risk of bleeding events and comparable efficacy in preventing S/SE compared to warfarin, presenting promising options for patients at high risk of bleeding. Rivaroxaban was superior to warfarin in preventing S/SE at both doses, while the bleeding risk remained comparable, suggesting that rivaroxaban is a preferred option for patients at lower risk of bleeding. For secondary outcome, we extracted all-cause mortality outcomes from 1,444,531 patients on 20 studies. SD apixaban (HR, 0.78; 95% CI, 0.71–0.87; *I*^2^, 88%) and SD dabigatran (HR, 0.75; 95% CI, 0.69–0.82; *I*^2^, 48%) were superior to warfarin in terms of mortality. The difference between warfarin and SD and LD edoxaban (HR, 0.54; 95% CI, 0.30–0.99; *I*^2^, 92%, and HR, 0.72; 95% CI, 0.56–0.92; *I*^2^, 60%, respectively) was also statistically significant, but results were based on fewer studies. There was no significant difference in mortality among the other comparisons (Figure S2). These results regarding mortality also reinforced the preference for SD apixaban, SD dabigatran, and SD edoxaban.

### 3.3. Sensitivity Analysis

**Figure 4:**
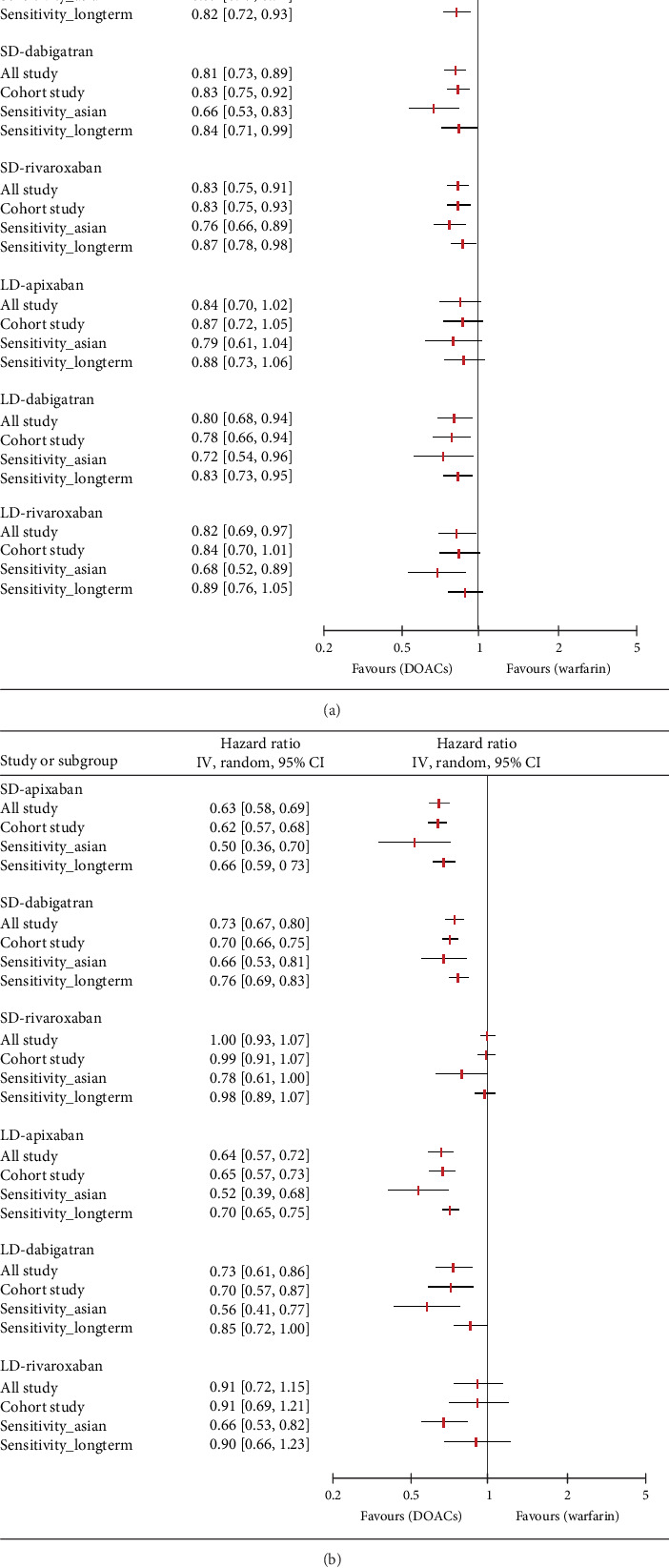
Sensitivity analysis: (a) stroke/systemic embolism; (b) major bleeding.

With the exception of edoxaban, for which there were insufficient relevant studies, we conducted a further analysis and sensitivity analysis. In a further analysis, which focused solely on observational studies, we found no meaningful changes compared to the primary analysis (Figure 4). The only exception was that there was no significant difference in the prevention of S/SE with LD rivaroxaban compared to warfarin (HR, 0.84; 95% CI, 0.70–1.01; *I*^2^, 65%). In a sensitivity analysis involving East Asian patients across 12 studies, all dose-specific DOACs showed lower HR values for S/SE than those observed in the primary analysis, with no changes in statistical significance. Similarly, for MB, HR values were lower in East Asian patients compared to the primary analysis that included all ethnicities. Notably, LD rivaroxaban significantly reduced the risk of MB in East Asians compared with warfarin in contrast to all ethnicities (HR, 0.66; 95% CI, 0.53–0.82; *I*^2^, 53%, respectively). In the other sensitivity analysis of 20 studies with more than 6 months of follow-up, both primary outcomes were generally consistent with HR values and statistical significance of the primary analysis. However, compared to warfarin, LD rivaroxaban did not significantly reduce the risk of S/SE risk (HR, 0.89; 95% CI 0.76–1.05; *I*^2^, 50%) and LD dabigatran did not show a significant difference from warfarin in MB incidence (HR, 0.85; 95% CI 0.72–1.00; *I*^2^, 67%). The number of studies and patients included in the sensitivity analyses is presented in Table S2.

### 3.4. Rank Probability

**Figure 5:**
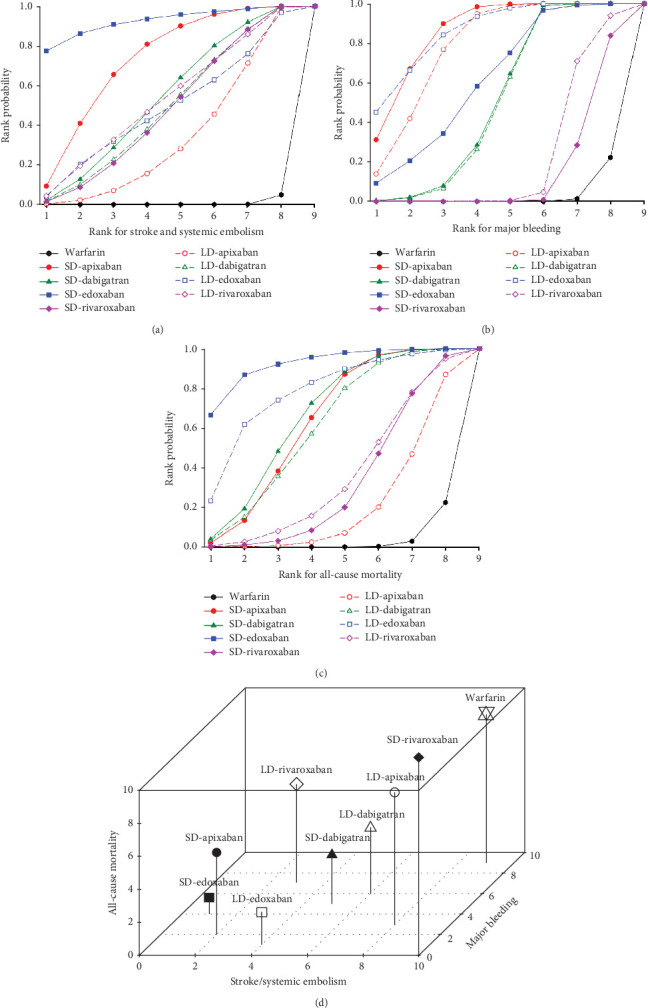
Rank probability: (a) rank probability for stroke/systemic embolism; (b) rank probability for major bleeding; (c) rank probability for all-cause mortality; (d) comprehensive rank. SD: standard dose, LD: low dose.

We presented the results of our network meta-analysis for S/SE using rank probability values (Figure 5(a)). SD edoxaban had the highest score (0.77 at Rank 1), indicating that SD edoxaban had the least S/SE risk among the groups. LD edoxaban (0.45 at Rank 1) had the highest score in the MB, indicating that the incidence of MB in patients taking LD edoxaban was lower than that of patients in the other treatment groups (Figure 5(b)). In terms of mortality, SD edoxaban was the most preferred (0.67 at Rank 1) (Figure 5(c)). SD apixaban (S/SE: second, MB: second), SD edoxaban (S/SE: first, MB: fourth), and LD edoxaban (S/SE: fourth, MB: first) had high ranks for both effectiveness and safety in the overall interpretation of primary outcomes. This result indicates that these dosing regimens have a lower incidence of S/SE and MB than the others.  Figure 5(d) shows the ranks including primary and secondary outcomes.

### 3.5. Publication Bias

Publication bias for primary outcomes was not found in the funnel plot, trim-and-fill method, Begg's test, and Egger's test (*p* > 0.05 for all analyses; Figure S3). This indicates that our review included a balanced number of both positive and negative studies regarding the primary outcome. In the mortality analysis, Begg's test revealed evidence of publication bias (*p* = 0.041) while Egger's test and the trim-and-fill method did not.

## 4. Discussion

RCTs, including clinical trials, are the gold standard for demonstrating the impact of interventions [51]. However, the patients enrolled in the RCT do not generally represent the patients treated in actual clinical practice [52–54]. This meta-analysis incorporating real-world data (RWD) and RCTs could provide a more comprehensive understanding of the effectiveness and safety of interventions in real-world clinical practice.

Meta-analyses, including RCTs and RWD, have been published to evaluate the benefits of DOACs in specific vulnerable populations with AF [55–57]. The appropriateness of dose titration is emphasized in these patients, and these patients are more likely to be taking low-dose direct-acting oral anticoagulants (LD-DOACs). In patients with low body weight, DOACs were more effective in preventing recurrent stroke than warfarin [55]. In a study of patients on dialysis, there was no statistically significant difference in the reduction of ischemic stroke and MB [56]. A network analysis of elderly patients aged 75 years and older reported no difference between DOACs in the incidence of thrombotic events, and the risk of intracranial hemorrhage was lower with apixaban, dabigatran, and edoxaban compared to rivaroxaban and warfarin [57].

In our network meta-analysis, which included RCTs and RWD with no restrictions on vulnerable groups, apixaban had different trends in the ARISTOTLE trial. The ARISTOTLE trial suggested that all doses of apixaban are more advantageous than warfarin in both S/SE and MB [58]. However, in our study, the rank probability results for effectiveness showed that the apixaban group exhibited the most significant difference in SD (second) and LD (eighth), among the DOACs. LD apixaban was not evaluated in its Phase 2 trial, and it was introduced in Phase 3 (ARISTOTLE) to address the potential over exposure in high-risk patients [59]. It is important to recognize that the population in Phase 3 was not representative of the real world. Only 428 (4.7%) of apixaban users received the low dose [20], but apixaban was reportedly prescribed at a low dose in 20.8% of patients in a real-world observational study [60]. In contrast, rivaroxaban was prescribed at a low dose in 21.7% of total patients in the real-world study, similar to the Rivaroxaban Once Daily Oral Direct Factor Xa Inhibition Compared with Vitamin K Antagonism for Prevention of Stroke and Embolism (ROCKET-AF)trial [21]. This discrepancy between clinical trial participants and real-world patients is a contributing factor to the differing trends observed for apixaban doses in this study.

Our study suggested that dabigatran showed superior effectiveness to warfarin in S/SE at both standard and low doses. These results are consistent with those reported in the other network meta-analysis study comparing the effectiveness and safety of DOACs according to renal function [61]. In a subanalysis of Randomized Evaluation of Long-term Anticoagulation Therapy) RE-LY, LD dabigatran was found to be associated with a 7%–15% reduction in S/SE compared to warfarin, though no statistical difference was observed [62]. Furthermore, the summary of product characteristics (SmPC) for dabigatran, as approved by the European Medicines Agency, recommends dose reduction only for patients at high bleeding risk, rather than dose adjustment based on renal function [63]. Our results provide support for the current dose adjustment strategy of dabigatran and suggest that LD dabigatran is the preferred option for safety as well as efficacy in patients aged 75 years or older; patients with gastritis, esophagitis, or gastroesophageal reflux disease; and patients at increased risk of bleeding, as outlined in the SmPC. Additionally, dabigatran showed more favorable outcomes than warfarin in terms of MB. Although these findings differ from those of large-scale dabigatran trial (RE-LY) [19], both the 150 mg and 110 mg showed superior effectiveness and safety to the warfarin group, supporting the current dose regimen.

Edoxaban was analyzed with the highest priority in the rank probability tests; however, the limited number of included studies warrants caution in interpreting the results. In the Effective Anticoagulation with Factor Xa Next Generation in Atrial Fibrillation–Thrombolysis in Myocardial Infarction 48 (ENGAGE-AF TIMI 48) trial, patients were randomly assigned to either the high-dose (60 mg) or low-dose group (30 mg) without any adjustment criteria, followed by dose halving for those with estimated creatinine clearance of 30–50 mL/min, body weight ≤ 60 kg [23]. In a subgroup analysis of the high-dose edoxaban group, the original dosing group (edoxaban 60 mg) had an advantage in the prevention of S/SE compared to warfarin, whereas the dose halving group (edoxaban 30 mg) had no significant difference [23]. It is consistent with our study results. Therefore, edoxaban can be considerable for use as intended, with both doses offering an advantage over warfarin in terms of MB.

SD rivaroxaban and LD rivaroxaban were superior to warfarin in preventing S/SE, but they did not significantly differ in terms of MB outcome [3]. It suggests that a new dose adjustment for rivaroxaban might be needed. Adjusting both standard and low doses could maintain the effectiveness of rivaroxaban while improving its safety profile. In real-world clinical practice, particularly in Asia, lower doses (15 mg instead of 20 mg and 10 mg instead of 15 mg) are often used [32, 64–66]. In the ROCKET-AF study, no significant difference from warfarin was found in the MB incidence [67]. Instead, some bleeding-related events (transfusion, hemoglobin ≥ 2 g/dL, and gastrointestinal bleeding) occurred more frequently with rivaroxaban than with warfarin [67]. For intracranial hemorrhage, subgroup analysis according to dose reported that only SD users showed favorable results, and LD users showed no significant difference from warfarin [21]. Based on the safety outcomes of the ROCKET-AF study and our study, it is not possible to conclude that rivaroxaban is superior to warfarin in terms of bleeding side effects. Therefore, the standard and low doses require new adjustment strategies to maintain effectiveness and enhance the safety profile.

In terms of S/SE outcomes, the apixaban and edoxaban groups demonstrated distinct trends depending on dose. SD groups of both drugs outperformed the warfarin group in S/SE prevention, while LD groups showed no significant difference. However, both apixaban and edoxaban, regardless of dose, were superior to the warfarin group in MB events. These divergent outcomes between doses for apixaban and edoxaban may be partially explained by pharmacokinetic differences. In contrast to dabigatran and rivaroxaban, which have dose adjustment strategies of reducing the dose by 25%, apixaban and edoxaban require a 50% reduction. It raises concerns about maintaining pharmacokinetic similarity after dose reduction in these two drugs. Previous studies reported that the lower apixaban concentration in the LD group are associated with reduced anti factor Xa activity [68–70]. Similar findings apply to edoxaban [71]. The ARISTOTLE study reported that the SD group exhibited roughly 25% higher exposure than the LD group [58]. The AVERROES study revealed a 20% difference in chromogenic anti–factor Xa assay results between doses, which was proportional to the plasma concentration [72]. In the high-dose group of ENGAGE-AF TIMI study, a dose halving resulted in a decrease in plasma concentration of the edoxaban by approximately 29% and a reduction in average anti–factor Xa activity by 25% [23]. In contrast, low-dose rivaroxaban was designed using pharmacokinetic modeling to ensure equivalent drug exposure between doses [73]. Postmarketing analysis of ROCKET-AF data confirmed that drug exposures remained similar between standard and low doses, as predicted by modeling [74]. The studies for dabigatran demonstrated no significant differences in concentration or AUC between the two doses [72, 75–77]. Comparing to other DOACs, apixaban exhibited larger concentration differences between standard and low doses. However, pharmacokinetic data alone cannot fully explain the potential shortcomings of dose adjustment approach. Globally, approximately 20% of DOAC prescriptions were estimated as the off-label LD use, with 31% of apixaban prescribed being underdosed [40]. This off-label use reflects the real-world prescribing patterns, where apixaban is often prescribed to vulnerable patients [47]. This approach further exacerbates the limitations of the current apixaban dose-adjustment strategy, since the off-label LD patients are at risk for reduced drug exposure compared to on-label LD patients [40, 78, 79]. The patient included in the ARISTOTLE study was not representative of real-world clinical practice, where off-label prescriptions are a common occurrence. Therefore, further research is crucial to develop a new dose adjustment regimen for apixaban that optimizes its effectiveness while maintaining its lower bleeding risk compared to warfarin. Particularly, in-depth studies are needed on the effects in patients with renal dysfunction, underweight patients, or in the elderly who are on apixaban dose adjustment.

Sensitivity analysis of the effectiveness of dose-specific DOACs in preventing S/SE did not differ statistically from the primary analysis. In addition, the lower HR for S/SE in Asians compared to that in all ethnicities was expected from previous studies [80, 81]. For MB, LD dabigatran and LD rivaroxaban did not reach statistical significance compared to warfarin in all ethnicities but showed statistical significance in the Asian analysis. Given the prescribing patterns of Asians, it was explained that LD-DOACs were more often underdosed (off-label use) in observational studies, resulting in a lower incidence of MB with LD-DOACs compared to all ethnicities, including non-Asians. According to the study by Yan et al., which analyzed the regional differences in the consumption pattern of each DOAC, rivaroxaban and apixaban contributed to the majority of DOAC consumption worldwide, while edoxaban accounted for more than 25% in East Asian countries [82]. However, it was difficult to perform a sensitivity analysis of edoxaban in this meta-analysis because of the small number of studies for edoxaban. Further meta-analysis of DOAC use in Asians, including edoxaban, are needed to conduct in the future.

Our study has several limitations that should be considered when interpreting our results. First, we employed a naïve pooling method, treating all study designs as equivalent and combined them directly. This approach did not assign additional weight to studies with higher quality methodology or adjust for potential biases [83]. While potentially limiting the precision of our estimates, this method avoids excluding valuable insights from RWD studies. Conversely, a meta-analysis restricted solely to RCTs might not fully capture the complex landscape of clinical practice. Therefore, our inclusion of RWD complements existing research by providing a more comprehensive picture. Second, clinical decisions regarding DOAC therapy in real-world practice should consider stroke prevention and MB reduction, making composite outcome analyses valuable. However, the meta-analysis in this study is difficult to interpret as a composite outcome analysis due to differing patient populations. Third, heterogeneity was inevitable due to the large number of studies included in the analysis. We attempted to minimize this by employing a random-effects model for the meta-analysis and examining the effects of race and duration of follow-up as potential modifiers. Additionally, we conducted further analyses on cohort studies to address heterogeneity related to study design. Despite this efforts, the *I*^2^ value remained largely unchanged, with some results showing only a slight decrease. High heterogeneity was observed exclusively in the LD groups (LD apixaban for S/SE, LD dabigatran for MB, and LD rivaroxaban for MB), indicating variability in dose reduction strategies among the studies included in this meta-analysis. An inherent limitation was that patient-level variables used as criteria for dose reduction (e.g., age, race, sex, and estimated glomerular filtration rate (eGFR)) were severely limited in availability. These clinical characteristics may serve as potential modifiers. However, heterogeneity is a natural aspect of real-world practice, suggesting that our findings reflect a diverse population in clinical settings, which this may have enhanced the external validity of our results.

## 5. Conclusion

Across RCTs and RWD studies, this network meta-analysis compared the effectiveness and safety of DOACs at different doses. All DOACs, regardless of dose, showed effectiveness and safety that were either superior to or not significantly different from warfarin. Among DOACs, SD apixaban, SD edoxaban, and LD edoxaban ranked highest in the comprehensive interpretation of primary outcomes using rank probabilities. Furthermore, dose-dependent effectiveness of apixaban highlights the need for further research to optimize dosing. Particularly, in-depth studies are required for patients with renal dysfunction, underweight individuals, and the elderly, who may require dose adjustments of DOACs.

## Figures and Tables

**Table 1:** Patient characteristics of studies included in the analysis.

**Study**	**Country**	**Study type**	**Data source**	**Treatment**	**Patient number**	**Age**	**Male ratio (%)**	**Follow-up period**	**CHA**_**2**_ **DS**_**2**_ **-VASc**	**HAS-BLED**
Connolly et al., [19]	Multinational	RCT (RE-LY)	NA	W	6022	71.6 (8.6)	63.3	2 years	NA	NA
				SD-D	6076	71.5 (8.8)	63.2			
				LD-D	6015	71.4 (8.6)	64.3			

Granger et al., [20]	Multinational	RCT (ARISTOTLE)	NA	W	8650	70 (63–76)	65.0	1.8 years	NA	NA
				SD-A	8664	69 (62–75)	65.6			
				W	402	70 (63–76)	65.0			
				LD-A	424	83 (81–85)	44.5			

Fox et al., [21]	Multinational	RCT (ROCKET AF)	NA	W	5640	71 (63–76)	64.6	707 days	NA	NA
				SD-R	5637	71 (63–76)	64.4			
				W	1476	79 (75–83)	44.1			
				LD-R	1474	79 (75–82)	45.0			

Hori et al., [22]	Japan	RCT (J-ROCKET AF)	NA	W	496	70 (65–75)	81.7	2.5 years	NA	NA
				LD-R	498	70 (64–75)	85.3			

Ruff et al., [23]	Multinational	RCT (ENGAGE AF-TIMI 48)	NA	W	7036	72 (64–78)	62.5	2.8 years	NA	NA
				SD-E	5251	72 (64–78)^b^	62.1^b^			
				LD-E	1784					
				LD-E^a^	5249	72 (64–78)	61.2			

Igamberdieva and Abdullaev, [24]	Uzbekistan	RCT	NA	W	36	NA	NA	18 months	NA	NA
				LD-R	73	NA	NA		NA	NA

Sørensen et al., [25]	Denmark	Cohort	National registry data	W	4237	72.3 (10.9)	57.6	131 days	2.0 (1.1)	2.6 (1.5)
				SD-D	765	79.6 (8.3)	63.3		1.9 (1.0)	2.2 (1.3)
				LD-D	830	67.9 (8.2)	47.3		2.3 (1.0)	3.4 (1.4)

Graham et al., [26]	United States	Cohort	National insurance data	W	67,207	NA	48	NA	NA	NA
				SD-D	56,576	NA	51	NA	NA	NA

Larsen et al., [27]	Denmark	Cohort	National registry data	W	35,436	72.4 (65–80)	58.8	1.9 years	2.2 (1.2)	2.8 (1.7)
				SD-A	6349	71.3 (66–77)	60.3		2.3 (1.2)	2.8 (1.6)
				SD-D	12,701	67.6 (62–72)	66.1		2.0 (1.1)	2.2 (1.4)
				SD-R	7192	71.8 (66–79)	56.9		2.2 (1.2)	2.8 (1.6)

Yao et al., [28]	United States	Cohort	Private insurance data	W	7695	73 (66–81)	53.2	0.5 years	2 (2–3)	4 (3–5)
				SD-A	6302	73 (66–81)^b^	53.1^b^		2 (2–3)	4 (3–5)
				LD-A	1393					
				W	14,307	70 (61–78)	59.6	0.7 years	2 (1–3)	3 (2–5)
				SD-D	13,048	70 (62–78)	60.3		2 (1–3)	3 (2–5)
				W	16,175	72 (64–80)	56.3	0.6 years	2 (2–3)	4 (2–5)
				SD-R	12,697	72 (64–79)	56.8		2 (2–3)	4 (2–5)

Lee et al., [29]	Korea	Cohort	EMR (single center)	W	549	72 (64–78)	62.3	1 year	3.3 (1.7)	NA
				SD-D	183	72 (65–77)^b^	61.2^b^		3.3 (1.6)	
				LD-D	366					

Nielsen et al., [30]	Denmark	Cohort	National registry data	W	38,893	71 (12.6)	59.6	2.3 years	2.4 (1.2)	3.0 (1.7)
				LD-A	4400	83.9 (8.2)	39.4		2.8 (1.1)	4.3 (1.5)
				LD-D	8875	79.9 (9)	46.3		2.7 (1.0)	3.8 (1.5)
				LD-R	3476	77.9 (13.5)	46.8		2.5 (1.2)	3.6 (1.8)

Lip et al., [31]	United States	Cohort	Pooled data of national and private insurance	W	78,554	73.5 (9.5)	56.3	159 days	2.9 (1.3)	3.6 (1.6)
				SD-A	78,554	73.3 (9.3)	55.8	127 days	2.9 (1.3)	3.6 (1.6)
				W	25,578	82.9 (7.1)	36.4	152 days	3.6 (1.3)	4.8 (1.5)
				LD-A	25,578	83.9 (7.5)	36.6	122 days	3.5 (1.3)	4.8 (1.5)
				W	31,531	71.6 (10.1)	60.7	156 days	2.7 (1.3)	3.3 (1.6)
				SD-D	31,531	71.4 (10.3)	59.7	123 days	2.6 (1.3)	3.3 (1.7)
				W	94,258	73.5 (9.4)	57.2	161 days	2.8 (1.3)	3.5 (1.6)
				SD-R	94,258	73.2 (9.2)	57.1	153 days	2.8 (1.3)	3.5 (1.6)

Lee et al., [32]	Taiwan	Cohort	National insurance data	W	16,000	78 (10)	52	1.4 years	3.0 (0.9)	4.0 (1.3)
				LD-R	14,971	78 (10)	52	1.2 years	3.0 (0.9)	4.0 (1.3)

Yu et al., [33]	Korea	Cohort	National insurance data	W	2840	68.3 (11.9)	63.0	5 months	NA	NA
				SD-E	2840	68.2 (9.5)	63.3			
				W	3016	72.6 (9.9)	53.3			
				LD-E	3016	72.8 (9.1)	52.0			

Cho et al., [34]	Korea	Cohort	National insurance data	W	10,409	70.6	45	15 months	2.5	3.38^c^
				SD-A	4661	70.7^b^	44^b^		2.4	3.37^c^
				LD-A	7841					
				SD-D	3138	70.6^b^	44^b^		2.5	3.38^c^
				LD-D	9455					
				SD-R	8601	70.6	44		2.5	3.38^c^

Graham et al., [35]	United States	Cohort	National insurance data	W	183,003	75.2	52.8	130 days	NA	NA
				SD-A	72,921	75.1	52.8			
				SD-D	86,293	75.1	52.9			
				SD-R	106,369	75.1	53.1			

Jeong et al., [36]	Korea	Cohort	EMR (Single center)	W	804	70.4 (10.2)	60.4	1 year	NA	3.4 (1.8)
				SD-R	390	71.4 (10.5)^b^	63.3^b^			3.3 (1.8)
				LD-R	414					

Park et al., [37]	Korea	Cohort	National insurance data	W	28,839	73.1 (9.4)	53.2	0.7 years	5.8 (1.5)	4.2 (1.1)
				SD-A	12,073	71.7 (9.4)	60.2		5.5 (1.5)	4.0 (1.1)
				SD-D						
				SD-E						
				SD-R						

Chan et al., [38]	Taiwan	Cohort	National insurance data	W	19,761	74.6 (10.7)	57.9	NA	2.6 (1.3)	3.2 (1.8)
				SD-A	3593	69.8 (6.6)	63.4		2.4 (0.8)	3.0 (1.0)
				LD-A	6359	78.3 (6.3)	53.6		2.8 (0.7)	4.0 (1.0)
				SD-D	2550	68.9 (4.6)	65.8		2.5 (0.5)	3.1 (0.6)
				LD-D	19,821	75.3 (4.7)	56.5		2.7 (0.5)	3.6 (0.7)
				SD-R	1914	70.1 (3.8)	64.5		2.4 (0.4)	3.1 (0.5)
				SD-E	1653	70.5 (10.7)	65.9		2.4 (1.1)	3.2 (1.5)
				LD-E	2924	77.1 (10.1)	52.4		2.8 (1.1)	3.8 (1.5)

Kohsaka et al. [39]	Japan	Cohort	Pooled data of EMR and national insurance	W	19,059	76.1 (11.9)	61.2	452	NA	3.8 (2.1)
				SD-A	10,213	76.1 (10.8)^b^	61.2	396 days		3.8 (1.9)
				LD-A	12,539					
				SD-D	1445	75.6 (10.3)^b^	62.0	823 days		3.8 (2.0)
				LD-D	6557					
				SD-E	3216	76.2 (10.8)^b^	61.1	263 days		3.8 (2.0)
				LD-E	9376					
				LD-R	8260	76.2 (10.6)	61.1	415 days		3.8 (1.9)

Chan et al., [40]	Taiwan	Cohort	EMR (multicenter)	W	2342	68.0 (13.4)	56	NA	2.4 (1.5)	2.9 (1.9)
				LD-A	799	75.3 (9.2)	60		3.0 (1.3)	3.3 (1.5)
				LD-D	1004	67.8 (7.6)	66		2.4 (1.2)	2.8 (1.4)
				LD-E	338	72.4 (9.8)	68		2.6 (1.2)	3.1 (1.5)

Han et al., [41]	Korea	Cohort	National insurance data	W	4774	75.5	50.3	NA	3.7	5.0
				LD-A	4774	75.5	50.6		3.8	5.0
				W	5221	73.9	55.1		3.6	4.7
				LD-D	5221	73.9	55.6		3.6	4.7
				W	5746	74.2	54.6		3.7	4.7
				LD-R	5,746	74.2	55.0		3.7	4.7

Wetmore et al. [42]	United States	Cohort	Pooled data of USRDS and national insurance	W	12,298	NA	61.7	479 days	4.5 (2.0)	3.0 (1.1)
				SD-A	2123	NA	60.8	341days	4.4 (4.4)	2.9 (2.1)
				LD-A	1881	NA	61.2	305 days	4.5 (4.5)	3 (2.1)

Rutherford et al., [43]	Norway	Cohort	National registry data	W	6650	82.9 (5.1)	50.1	19.9 months	2.9 (1.0)	4.7 (1.4)
				SD-A	7631	80.8 (4.6)	50.9	12.7 months	2.8 (1.0)	4.3 (1.3)
				LD-A	6155	85.6 (5.3)	38	11.6 months	3.0 (1.1)	4.7 (1.4)
				SD-D	931	78.0 (3.5)	58.5	24.4 months	2.6 (1.0)	3.9 (1.3)
				LD-D	2926	83.0 (4.9)	44.2	17.8 months	2.8 (0.9)	4.4 (1.4)
				SD-R	3630	81.0 (4.8)	50.1	19.0 months	2.7 (1.0)	4.2 (1.3)
				LD-R	2,478	84.4 (5.4)	43.5	16.2 months	3.0 (1.0)	4.6 (1.4)

Halvorsen et al., [44]	Norway Denmark Sweden	Cohort	National registry data	W	42,672	72.1	60.7	9.7 months	1.8 (1.0)	3.0 (1.7)
				SD-A	42,672	72.2	60.2		1.8 (1.0)	3.0 (1.7)
				W	18,794	84.5	42.6		2.4 (1.0)	4.3 (1.5)
				LD-A	18,794	84.5	42.0		2.4 (1.0)	4.3 (1.5)
				W	18,701	67.9	67.6		1.6 (1.1)	2.3 (1.5)
				SD-D	18,701	67.9	67.3		1.6 (1.0)	2.3 (1.5)
				W	23,703	72.7	58.5		1.9 (1.0)	3 (1.7)
				SD-R	23,703	72.5	58.6		1.9 (1.0)	3 (1.7)

Perreault et al., [45]	Canada	Cohort	Provincial insurance data	W	14,700	81.5 (9.1)	41.8	1 year	3.3 (1.3)	4.0 (1.4)
				LD-A	3829	82.2 (7.9)	41.1		3.4 (1.3)	4.2 (1.3)
				W	14,700	80.2 (9.1)	44.2		3.3 (1.3)	3.9 (1.4)
				LD-D	1929	80.2 (7.7)	43.2		3.4 (1.3)	4.0 (1.3)
				W	14,700	80.4 (9.1)	43.9		3.0 (1.3)	4.0 (1.4)
				LD-R	1,718	80.7 (7.8)	43.0		3.3 (1.2)	4.0 (1.3)

Di et al., [46]	China	Cohort	EMR (single center)	W	227	77.4 (0.6)	56.8	2 years	3.2 (0.2)	NA
				LD-D	226	77.3 (0.5)	58.4		3.2 (0.2)	
				LD-R	227	77.4 (0.6)	59.5		3.1 (0.2)	

Lin et al., [47]	United States	Cohort	Pooled data of national and private insurance	W	250,995	78.1 (7.3)	49.7	165.8 days	4.7 (1.7)	2.3 (0.7)
				SD-A	250,995	78.1 (7.4)	49.9		4.7 (1.7)	2.3 (0.7)

Albabtain et al., [48]	Saudi Arabia	Cohort	EMR (single center)	W	164	74 (66–81)	60.4	49.5 months	NA	NA
				LD-R	149	75 (70–80)	52.4	50.0 months		

Perreault et al., [49]	Canada	Cohort	Provincial insurance data	W	3335	80.1 (10.8)	45.2	320 days	3.9 (1.5)	3.3 (1.5)
				SD-A	2082	80.2 (7.8)	46.1	365 days	4.2 (1.3)	3.3 (1.2)
				W	3335	80.4 (10.2)	45.7	320 days	4.2 (1.4)	3.3 (1.4)
				SD-R	1,064	79.1 (8.2)	47.7	365 days	3.9 (1.5)	3.4 (1.4)

Tsai et al., [50]	Taiwan	Cohort	EMR (multicenter)	W	174	76.1 (10.0)	45.4	NA	3.4 (1.5)	2.6 (1.2)
				SD-A	128	78.8 (6.8)	45.3		4.0 (1.4)	2.9 (1.1)
				LD-A	215	82.1 (7.0)	46.0		4.4 (1.4)	3.2 (1.2)

*Note:* SD-D: dabigatran 150 mg bid; LD-D: dabigatran 110 mg bid; SD-R: rivaroxaban 20 mg qd, LD-R: rivaroxaban 15 mg qd, SD-A: apixaban 5 mg bid, LD-A: apixaban 2.5 mg bid, , SD-E: edoxaban 60 mg qd, LD-E: edoxaban 30 mg qd.

Abbreviations: EMR: electronic medical record, NA: not available, USRDS: United States Renal Data System, W: warfarin.

^a^E 30 of the low-dose group.

^b^DOACs without dose classification.

^c^Modified HAS-BLED.

## Data Availability

The data supporting this meta-analysis are from previously reported studies and datasets, which have been cited. The processed data are available from the corresponding author upon request.

## Supporting Information

Supporting Information 1 Table S1: PRISMA 2020 checklist. Table S2: Patient number of DOACs included in each analysis.

Supporting Information 2 Figure S1: Assessment for the risk of bias in the eligible studies: (A) RoB; (B) RoBANS. Figure S2: Forest plot of hazard ratio for mortality: (A) standard dose; (B) low dose. Figure S3: Assessment of publication bias in studies: (A) funnel plot and trim and fill; (B) Egger's test; (C) Begg's test.
