# Supplementary material for: Effectiveness and Safety of Dose-Specific DOACs in Patients With Atrial Fibrillation: A Systematic Review and Network Meta-Analysis
Source: Cardiovasc Ther. 2025 Jan 6;2025:9923772. doi: 10.1155/cdr/9923772 (PMC11729532; doi:10.1155/cdr/9923772)
Supplement: Supporting Information 2 — Figure S1: Assessment for the risk of bias in the eligible studies: (A) RoB; (B) RoBANS. Figure S2: Forest plot of hazard ratio for mortality: (A) standard dose; (B) low dose. Figure S3: Assessment of publication bias in studies: (A) funnel plot and trim and fill; (B) Egger's test; (C) Begg's test. [file 9923772.f2.docx]

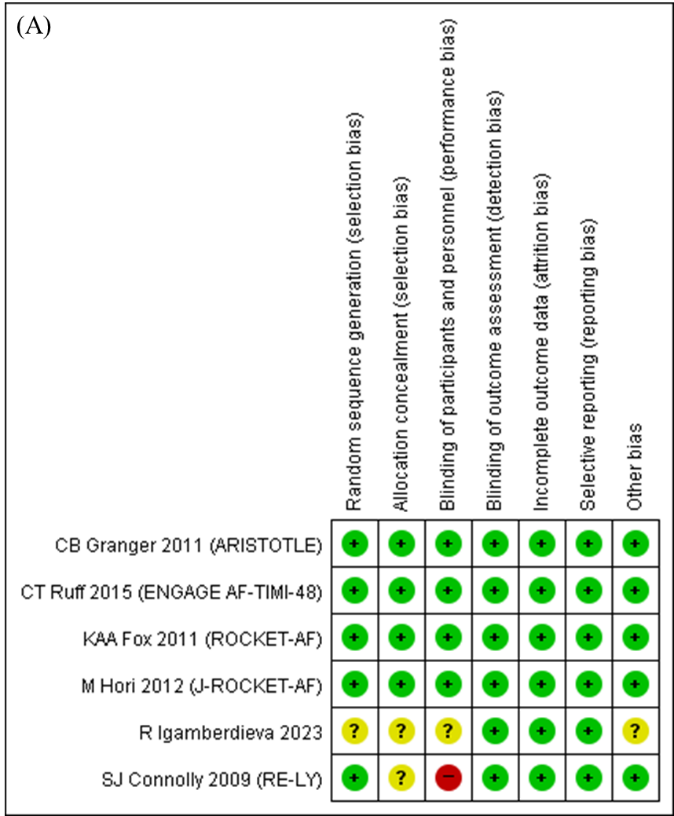

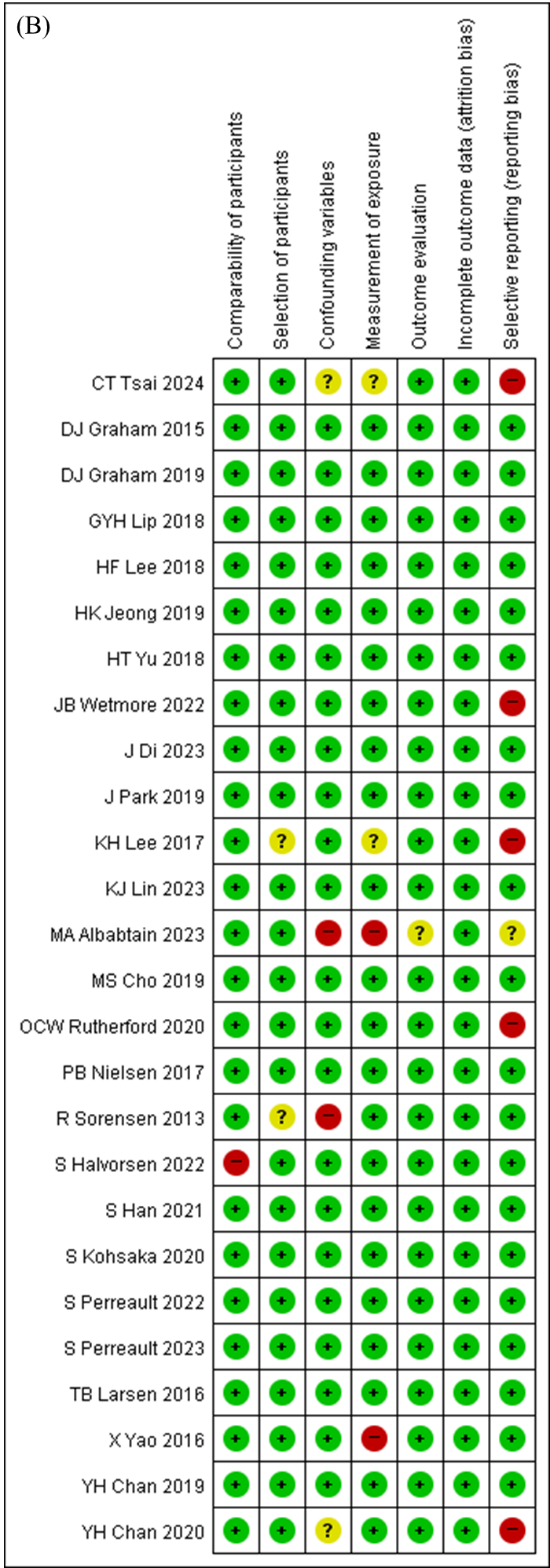


**Supplementary Figure 1.** Assessment for the risk of bias in the eligible studies: (A), RoB; (B), RoBANS


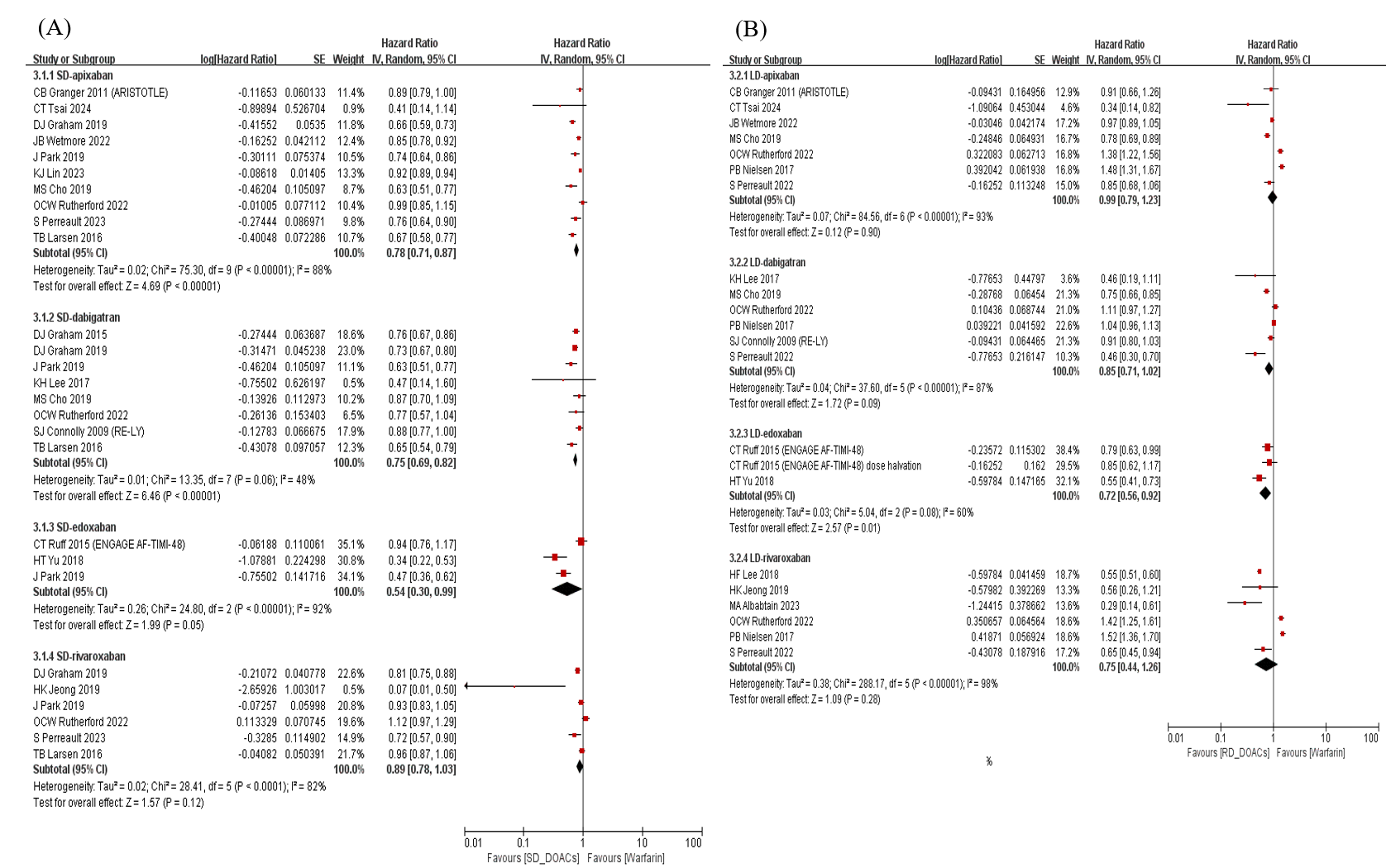
**Supplementary Figure 2**. Forest plot of hazard ratio for mortality: (A), Standard-dose; (B), Low-dose


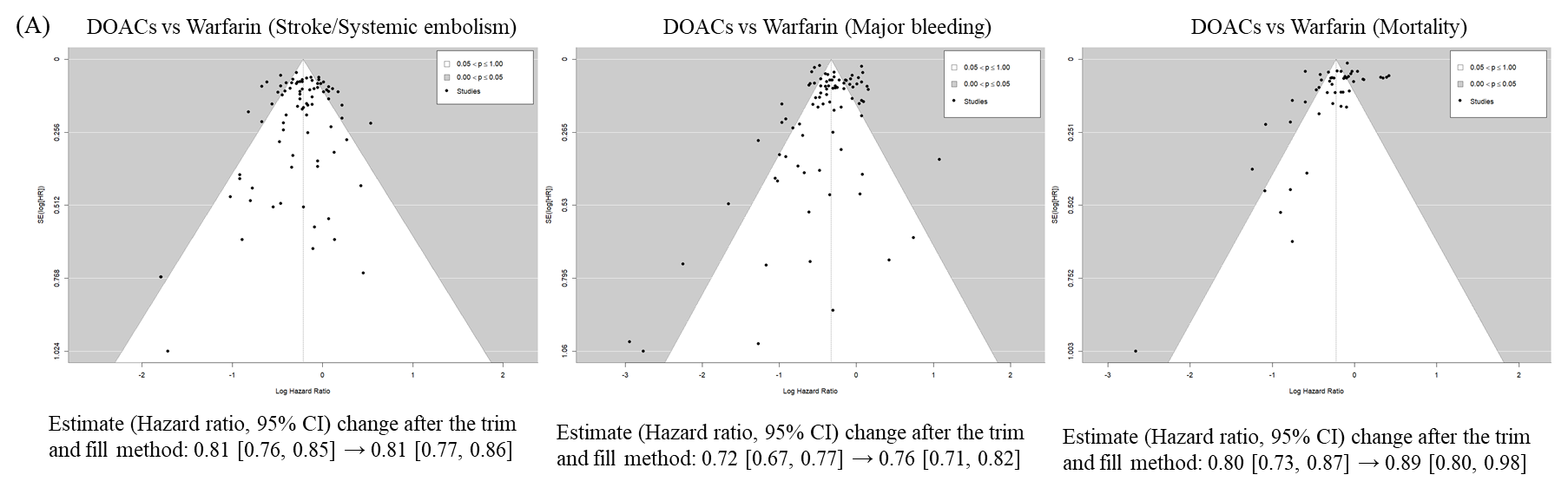


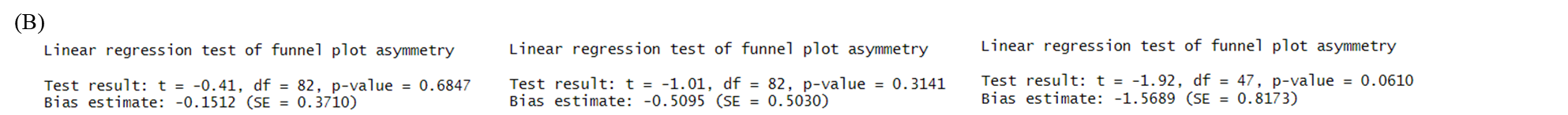


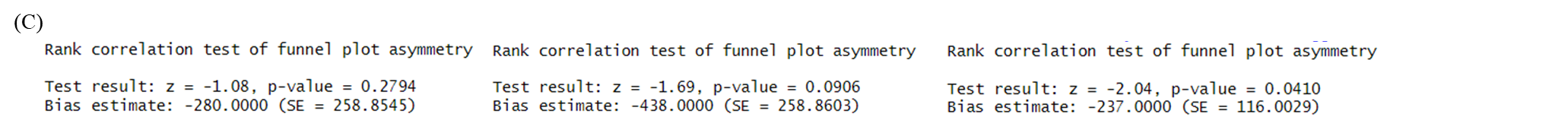


**Supplementary Figure 3.** Assessment of publication bias in studies: (A), Funnel plot & trim-and-fill; (B), Egger’s test; (C), Begg’s test
